# Fosfomycin susceptibility of uropathogens at Charlotte Maxeke Johannesburg Academic Hospital

**DOI:** 10.4102/sajid.v35i1.173

**Published:** 2020-10-26

**Authors:** Lesego M. Mothibi, Norma N. Bosman, Trusha Nana

**Affiliations:** 1 Department of Clinical Microbiology and Infectious Diseases, Faculty of Health Sciences, University of Witwatersrand, Parktown, South Africa; 2 National Health Laboratory Services, Chris Hani Baragwanath Academic Hospital, Johannesburg, South Africa; 3 National Health Laboratory Services, Charlotte Maxeke Johannesburg Academic Hospital, Johannesburg, South Africa

**Keywords:** fosfomycin susceptibility, urinary tract infections, uropathogens, multidrug-resistant organisms, ciprofloxacin resistance, hospital, ambulatory

## Abstract

**Background:**

Multidrug-resistant uropathogens are becoming widespread both in community and hospital setting. Safe yet effective treatments are a priority. Fosfomycin is an antibacterial that displays good activity against most bacteria causing urinary tract infections (UTIs), including multidrug-resistant bacteria. The aim of this study was to evaluate fosfomycin susceptibility for uropathogens isolated from a microbiology laboratory at a tertiary academic hospital. In addition, this was compared to the susceptibility of other oral antimicrobials.

**Methods:**

We conducted a retrospective analysis of laboratory reports for uropathogens isolated at Charlotte Maxeke Johannesburg Academic Hospital from September 2015 to August 2017. Antimicrobial susceptibility testing of the isolates was performed using the Kirby–Bauer disk diffusion method or the Vitek^®^ 2 system according to the Clinical and Laboratory Standards Institute.

**Results:**

Overall susceptibility of fosfomycin for the 4700 Enterobacteriaceae isolates was 95.7%; 95% confidence interval (CI) 95.1–96.2. The overall susceptibility for fosfomycin against the gram-positives was 98.6%. There were 37.9% multidrug-resistant Enterobacteriaceae (MDRE) isolated during the study period. Fosfomycin displayed activity against 94.4% of extended-spectrum β-lactamase (ESBL) producers and 90.7% for carbapenem-resistant Enterobacteriaceae (CRE). None of the methicillin-resistant *Staphylococcus aureus* and vancomycin-resistant *Enterococcus* isolates tested was fosfomycin resistant. The overall in vitro susceptibility was significantly higher for fosfomycin (*p* = 0.0001) compared to amoxicillin/clavulanic acid, cephalexin, cefuroxime, ciprofloxacin, trimethoprim/sulfamethoxazole and nitrofurantoin.

**Conclusion:**

This study confirmed the high susceptibility of fosfomycin against UTI pathogens isolated at our institution. In an era of increasing antimicrobial resistance, fosfomycin represents a potential option for the treatment of UTIs at Charlotte Maxeke Johannesburg Academic Hospital.

## Introduction

Urinary tract infections (UTIs) have become the most common bacterial infections, equally found in the community and in hospitals.^1^ Antibiotics such as ciprofloxacin, trimethoprim/sulfamethoxazole and nitrofurantoin were used successfully as therapy for UTIs.^2^ However, because of the increased drug resistance to these antibiotics, the choice of empiric antimicrobials for UTIs has become more challenging.^3,4^ As a result, there is a growing need to find novel treatment options or to re-examine old antibiotics such as fosfomycin.

Fosfomycin is a broad-spectrum antimicrobial agent that inhibits the synthesis of the bacterial cell wall of both gram-negative and gram-positive bacteria.^5^ The Infectious Diseases Society of America (IDSA) 2010 guidelines on the treatment of uncomplicated UTIs recommend fosfomycin 3 g oral single-dose as one of the first-line treatment options.^6^ Fosfomycin displays good activity against most bacteria-causing UTIs, including multidrug-resistant (MDR) organisms.^5^ It is well tolerated with minimal side effects, such as diarrhoea and headache, and is not contraindicated in pregnancy.^5^ Some clinical trials have shown that a single 3 g dose of fosfomycin is as effective as a 7-to-a-10-day regimen using other oral antibiotics to treat uncomplicated cystitis.^5^ The findings of a more recent open-label randomized trial suggest that fosfomycin efficacy for acute uncomplicated UTI in comparison to that of nitrofurantoin is lower.^7^

The oral formulation of fosfomycin has only recently become available at our centre. Susceptibility testing for fosfomycin on urinary isolates has since been introduced at Charlotte Maxeke Johannesburg Academic Hospital (CMJAH) microbiology laboratory. Consequently, there was a lack of information regarding the susceptibility rates of fosfomycin amongst uropathogens isolated at our centre. In this study, we aimed to analyse retrospective data for fosfomycin susceptibility of gram-negative and gram-positive bacteria isolated from urine samples and to compare its activity with that of other oral antimicrobials usually used to treat lower UTIs. Furthermore, the burden of MDR uropathogens was also established. This information will assist in determining the role of oral fosfomycin at our centre. In addition, it will help with the formulation of guidelines for therapy of lower UTIs locally.

## Methods

### Study design

This is a retrospective laboratory-based study undertaken at CMJAH microbiology laboratory. Charlotte Maxeke Johannesburg Academic Hospital is a 1088-bed university hospital. Charlotte Maxeke Johannesburg Academic Hospital offers secondary, tertiary and quaternary services, as well as outpatient clinics. It also serves as a referral centre for several hospitals.

Antimicrobial susceptibility testing (AST) data for uropathogens isolated at CMJAH from September 2015 to August 2017 were used for analysis. Furthermore, the burden of MDR uropathogens for specific high-risk units for multidrug-resistant organisms (MDROs) was established. The data for this study were retrieved from the National Health Laboratory Service (NHLS) Corporate Data Warehouse (CDW).

### Definitions

*Multidrug resistance* was defined as acquired non-susceptibility to at least one agent in three or more antimicrobial classes.^8^

*AmpC-β-lactamase producers (Amp C)*: Organisms resistant to cefoxitin were considered to be Amp-C producers.

*Extended-spectrum β-lactamases (ESBLs)*: Organisms resistant to ceftriaxone or ceftazidime were considered to be ESBL producers.

*Carbapenem-resistant Enterobacteriaceae (CRE)*: Organisms resistant to ertapenem were considered to be carbapenem resistant.

### Sample size

In total 8906 bacterial isolates were included in the study. Repeat bacteria cultured from the same patient were considered to be duplicate organisms representing a single infection if isolated within 2 weeks and were removed from the analysis.^9^ Fosfomycin results were not available for all the Enterobacteriaceae in the data retrieved from the CDW. In addition, fosfomycin susceptibility testing of gram-positive bacteria was performed on 76 selected non-duplicate isolates identified in the microbiology laboratory between June 2017 and August 2017.

### Bacterial culture and identification

The microbiological diagnosis of a UTI requires ≥10^5^ colony-forming units (CFU/mL) of a single microorganism in a midstream collected urine, or ≥10^4^ CFU/mL of a single microorganism in catheter samples, or any bacterial growth in urine obtained by suprapubic route.^10^ Bacterial culture and identification of urine specimens were performed according to the CMJAH Microbiology Laboratory Urine Standard Operating Procedures. Urine specimens were inoculated on 5% blood agar and MacConkey agar plates and incubated overnight at 35°C–37°C. The bacterial species identification was performed using the API^®^ 20E kit (bioMérieux SA, France) or the Vitek^®^ 2 system (bioMérieux SA, France).

### Antimicrobial susceptibility testing

Routine AST of the isolates was performed using the disk diffusion method or the Vitek^®^ 2 system (bioMérieux SA, France) according to the Clinical and Laboratory Standards Institute (CLSI).^11,12,13^ Fosfomycin susceptibility was determined by the disk diffusion method. The plates were incubated aerobically at 35°C–37°C for 18–24 h. The results were interpreted according to the CLSI guidelines breakpoints for the corresponding year.^11,12,13^ The CLSI provides fosfomycin interpretive breakpoints for *Escherichia coli* and *Enterococcus faecalis* only. The fosfomycin interpretive breakpoints for *E. coli* were used to interpret fosfomycin susceptibility for other Enterobacteriaceae, whilst the interpretive breakpoints for *E. faecalis* were used for gram-positive bacteria.^2^ For Enterobacteriaceae, CLSI interpretative breakpoints for cefazolin were used to predict susceptibility to cephalexin. Two bacterial strains were used for quality control (QC) of AST: *E. coli* (American Type Culture Collection [ATCC] 25922) and *Staphylococcus aureus* (ATCC 25923).^11,12,13^ Quality control was performed weekly.

### Data analysis

Fosfomycin and other oral antibiotic susceptibility results were reported as percentage susceptible. The difference between the percentage of isolates susceptible to fosfomycin and other oral antibiotics was calculated using 95% confidence intervals (CIs) and *p* values.

The 95% CI was calculated using the Poisson distribution. A *p* < 0.05 was considered statistically significant. All statistical analyses were conducted using STATA, version 14 (StataCorp, USA).

### Ethical consideration

The study was accepted by the Human Research Ethics Committee (Medical) of the University of the Witwatersrand, approval number M170935.

## Results

### Distribution of isolated uropathogens from Charlotte Maxeke Johannesburg Academic Hospital

The distribution of isolated uropathogens from CMJAH is shown in Table 1. From September 2015 to August 2017, a total of 9083 (21.9%) urines were culture positive. The predominant causative organisms were *E. coli, Klebsiella pneumoniae* and *E. faecalis*.

**TABLE 1:** Distribution of uropathogens isolates from Charlotte Maxeke Johannesburg Academic Hospital.

Uropathogens	*N*	%
**Total number of specimens†**	**41 446**	**-**
**Total number of specimens with uropathogens**	**9083**	**-**
**Total gram-negative bacilli isolated**	**7395**	**81.4**
*E. coli*	4142	56.0
*Klebsiella pneumoniae*	1252	16.9
*Klebsiella oxytoca*	98	1.3
*Proteus mirabilis*	475	6.4
*Enterobacter* species	314	4.2
*Citrobacter* species	129	1.7
*Pseudomonas aeruginosa*	364	4.9
*Acinetobacter baumannii*	241	3.2
Others	380	5.1
**Total gram-positive cocci isolated**	**1511**	**16.6**
*S. aureus*	143	9.5
*S. saprophyticus*	12	0.8
*E. faecalis*	792	52.4
*E. faecium*	182	12.1
*S. agalactiae*	227	15.0
Others	155	10.3
**Total *Candida* species**	**177**	**2.0**
*Candida albicans*	97	54.8
Non-*albicans Candida* species	80	45.2

† , Urine samples from September 2015 to August 2017 (study period).

### Fosfomycin susceptibility testing results

**FIGURE 1:**
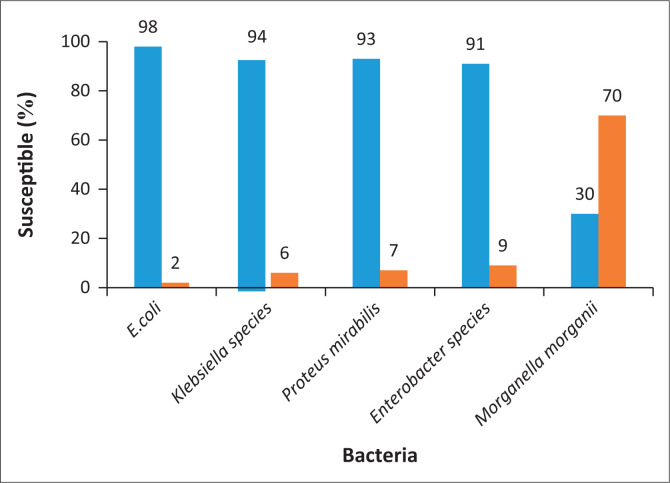
Antimicrobial susceptibility of urinary Enterobacteriaceae to fosfomycin.

Fosfomycin susceptibility testing results were available for 4700 Enterobacteriaceae. The overall susceptibility of fosfomycin for the Enterobacteriaceae was 95.7%; 95% CI: 95.1–96.2; *p*-value of 0.0001. Fosfomycin activity against *E. coli* was 98.1%. Other members of the Enterobacteriaceae family in this current study also showed high susceptibility to fosfomycin except for *Morganella* species (Figure 1).

**FIGURE 2:**
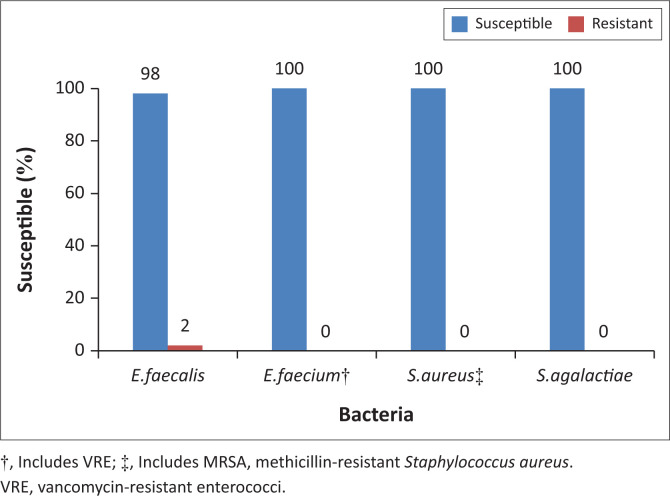
Antimicrobial susceptibility of urinary gram-positive cocci to fosfomycin.

A breakdown of the 76 gram-positive organisms tested for fosfomycin susceptibility demonstrated 47 (61.8%) *E. faecalis*, 15 (19.7%) *Enterococcus faecium*, 7 (9.2%) *S. aureus*, 4 (5.3%) *Streptococcus agalactiae* and 3 (3.9 %) *Staphylococcus saprophyticus* isolates. As *S. saprophyticus* is intrinsically resistant to fosfomycin, these isolates were removed from the analysis. The overall susceptibility for fosfomycin for the gram-positives was 98.6%. All the *S. aureus* isolates tested were susceptible to fosfomycin. Fosfomycin activity against enterococcal species, including the vancomycin-resistant enterococci (VRE) (*n* = 3) strains, was excellent. A single *E. faecalis* isolate was resistant to fosfomycin (Figure 2).

### Multidrug-resistant rates

**FIGURE 3:**
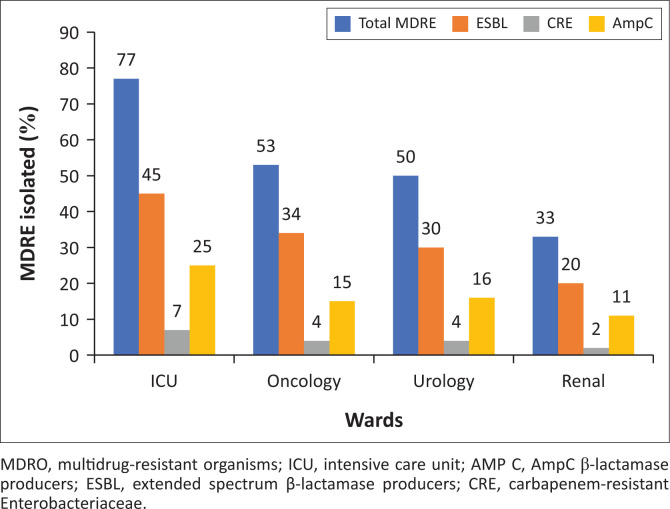
Multidrug-resistant enterobacteriaceae urinary isolates at high risk units for MDRO.

There were 2520 (37.9%) multidrug-resistant Enterobacteriaceae (MDRE) isolated during the study period. Extended-spectrum β-lactamase production was identified in 23.2% (*n* = 1545) of the Enterobacteriaceae (Table 2). The majority of ESBL producers were *K. pneumoniae* 636 (41.2%) and *E. coli* = 623 (40.3%) (data not shown). Overall, 24 (17%) of *S. aureus* isolates were methicillin-*resistant Staphylococcus aureus* (MRSA) and 11 (1.1%) of the *Enterococcus* species isolates were VRE (data not shown). The majority of MDRE were isolated from patients admitted to ICUs (Figure 3).

**TABLE 2:** Percentage of multidrug-resistant Enterobacteriaceae urinary isolates.

MDRE	*N*	%
Total number of Enterobacteriaceae	6652	-
Total number of MDRE	2520	37.9
ESBL	1545	23.2
AmpC	815	12.3
CRE	160	2.4

MDRE, multidrug-resistant Enterobacteriaceae; AMP C, AmpC β-lactamase producers; ESBL, extended spectrum β-lactamase producers; CRE, carbapenem-resistant Enterobacteriaceae.

For the subset of MDRE with fosfomycin results available, the fosfomycin susceptibility was: 94.4% for ESBL producers and 90.7% for CRE. All the MRSA and VRE isolates tested showed 100% susceptibility to fosfomycin (data not shown).

### Fosfomycin susceptibility compared with other oral agents used to treat Enterobacteriaceae causing urinary tract infections

**FIGURE 4:**
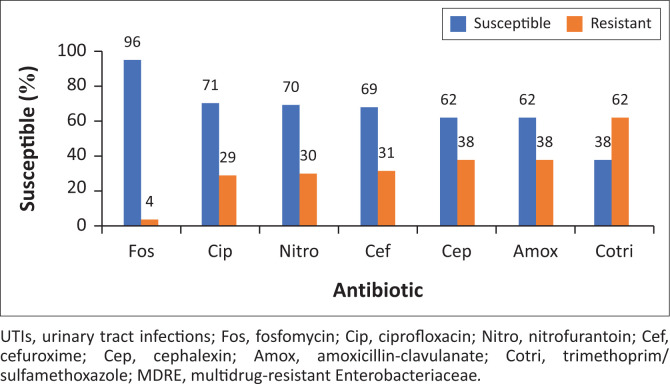
Antimicrobial susceptibility of fosfomycin compared with other oral antibiotics used to treat Enterobacteriaceae causing urinary tract infections (including multidrug-resistant Enterobacteriaceae [MDRE]).

In the present study, fosfomycin showed the highest susceptibility (95.7%, 95% CI: 95.1–96.2) against the tested Enterobacteriaceae. There was a statistically significant difference between the susceptibility rates of fosfomycin (*p* = 0.0001) and all the other oral agents tested. Ciprofloxacin, cefuroxime and nitrofurantoin susceptibility rates were modest; however, they were much lower compared to that of fosfomycin. The lowest susceptibility was seen with trimethoprim/sulfamethoxazole (38.35%, 95% CI: 37.2–39.5) (Figure 4).

## Discussion

The key finding of this study is that fosfomycin shows outstanding in vitro activity against uropathogens, including MDR isolates. Overall, the susceptibility of fosfomycin against Enterobacteriaceae was 95.7%. Fosfomycin activity against *E. coli* was higher than that of other Enterobacteriaceae. Significant susceptibility rates to fosfomycin were also found for *K. pneumoniae, P. mirabilis* and the tested gram-positive isolates. These findings are similar to those reported in the literature.^4,14,15^ *Morganella* species strains displayed significantly reduced susceptibility compared to other Enterobacteriaceae (70% resistance rates). This is in keeping with other studies, where fosfomycin-resistant rates for *Morganella morganii* range between 75% and 100%.^16,17^ *M. morganii* is regarded as being intrinsically resistant to fosfomycin.^18^ A study by Ito et al.^19^ detected fosA alleles in more than 90% of the *M. morganii* isolates. Production of fosfomycin-inactivating enzyme FosA is encoded by some clinically relevant gram-negative species and contributes to intrinsic fosfomycin resistance.^19^

The overall susceptibility of fosfomycin for gram-positives in this study was 98.6%. The most frequent gram-positive uropathogens were *Enterococcus* species, which were highly susceptible (98%) to fosfomycin. Research by Sultan et al.^20^ found that fosfomycin was highly effective against *Enterococcus* species and *Staphylococcus* species including VRE and MRSA. Fosfomycin is active against *S. aureus* biofilm production.^21^ Bacterial biofilm can play an important role in recurrent UTIs and resistance to antimicrobial agents.

Fosfomycin also demonstrated good activity against UTIs caused by MDRO. In this current study, the susceptibility of fosfomycin for MDRE causing UTIs was 90.7% (CRE), 92.3% (AmpC) and 94.4% (ESBL). Comparable to this study, a systematic review of *E. coli* ESBL susceptibility to fosfomycin revealed susceptibility rates of more than 81%.^22^ All MRSA and VRE tested in this research were susceptible to fosfomycin. Because of the high fosfomycin susceptibility rates against uropathogens in our setting, fosfomycin is an attractive choice of treatment for outpatients and hospitalised patients with suspected or culture-confirmed MDR (MDRE, VRE and MRSA) UTIs. A significant pharmacological benefit of fosfomycin is that it can be administered orally. The use of fosfomycin could prevent admission for the treatment of MDR UTIs or decrease the length of hospital stay by allowing substitution of oral for intravenous therapy. Fosfomycin could also prove useful for the treatment of UTIs in circumstances where therapy with conventional antibiotics was unsuccessful or is contraindicated. Furthermore, the use of aminoglycosides may be retained for MDR *A. baumannii* and *P. aeruginosa* treatment.

Studies have demonstrated the clinical efficacy of fosfomycin as therapy for lower UTIs caused by both susceptible and MDR uropathogens. A report by Neuner confirmed microbiological cure rates in 71% of patients with UTIs caused by VRE and ESBL producers. The study population included patients with co-morbidities such as diabetes mellitus, solid-organ transplant recipients and chronic kidney disease.^15^ Another study by Nagel et al.,^23^ compared clinical outcomes of fosfomycin in the hospital setting for patients with susceptible and MDR UTIs. A cure rate of 95% was documented. The majority of patients had UTIs caused by VRE (45%).^23^ A cohort study of hospitalised patients treated with oral fosfomycin for uncomplicated or complicated UTIs showed favourable clinical outcomes, with cure rates of 75%–90% for the different cohorts studied. Their study included patients with comorbidities and an equal proportion of community-acquired and nosocomial infections in the cohorts studied.^24^ A multinational study comparing a 5-day course of nitrofurantoin to a single 3-g dose of fosfomycin for uncomplicated UTIs in non-pregnant females showed clinical and microbiological cure rates of 58% and 63%, respectively, for the fosfomycin group.^19^ These were both significantly lower than that of the nitrofurantoin-treated group. Based on the findings of this study, further well-designed clinical trials assessing the role of fosfomycin is required.^25^

Globally, the susceptibility rates of fosfomycin have continued to be quite stable since its introduction. Studies performed in three European countries, where parenteral and oral fosfomycin has been used since the 1970s, showed no significant decrease in fosfomycin susceptibility.^26^ Fosfomycin exhibits a low level of cross-resistance to other antimicrobial agents because of its unique mechanism of action.^14^

Enterobacteriaceae are the common aetiological agents of UTIs.^3,4^ Our epidemiology is in keeping with the existing literature, where the majority of bacterial isolates were gram-negative bacilli (81.4%). The predominant causative organisms in this current study were *E. coli, K. pneumoniae* and *E. faecalis*. Even though gram-negative bacteria are the main causative organisms for complicated and uncomplicated UTIs, gram-positive bacteria are also important uropathogens.^3,4^ *S. saprophyticus, E. faecalis*, and *S. agalactiae* are the common cause of community-acquired UTIs in pregnant women and the elderly. *E. faecalis* and *E. faecium* are the third leading cause of nosocomial UTIs.^27^ The ratio of *E. faecalis* to *E. faecium* in this current study was 4:1. The incidence of UTIs caused by *E. faecalis* has escalated gradually and now outnumbers *E. faecium* UTIs.^27^

Rates of both nosocomial and community-acquired UTIs caused by MDR bacteria are escalating.^3,5,28^ In this study, 37.9% of Enterobacteriaceae were MDR. The ESBL phenotype resistance pattern was the common MDR pattern detected. β-Lactamase producers can express resistance to other classes of antibiotics.^29^ The increasing rate of resistance to multiple antibiotic classes and the high rate of MDRE may lead clinicians to use more carbapenems and colistin. Colistin is a potentially nephrotoxic antibiotic and is associated with neurological toxicity.^30^ Carbapenems and colistin are parenteral antibiotics and necessitate patient hospitalisation, which is linked to substantial financial burdens. Systemic exposure to broad-spectrum antibiotics is also associated with increased selection of MDR pathogens.^31^ This study shows that MRSA (17%) isolates are common uropathogens in our institution (data not shown). A comparable MRSA rate (14%) was reported in a study by Rajuduraipandi.^32^ In contrast, higher rates of 80.9% in Indore and 50.4% in eastern Uttar Pradesh were reported.^33,34^ This variability could be because of the difference in study design, population, antibiotic prescribing habits and geographic distribution.

This study did not look at the risk factors associated with MDRE. Rather, we focused on establishing the burden of MDRE uropathogens in selected wards. Previous studies have acknowledged that prior fluoroquinolone use within 3 months, a hospital stay for at least 48 h, comorbidities, an indwelling urinary catheter, and urological procedures within the past 3 months are independent risk factors linked with MDRE UTIs.^35^ The distribution of MDRE UTIs in the selected high-risk wards is shown in Figure 3. The majority of MDRE UTIs were from patients admitted to ICU (77%). Indwelling urinary catheters, the length of ICU admission and selective pressure from the use of antimicrobial agents are the most likely risk factors for the high rates of MDRE in ICU. The findings emphasise the significance of antibiotic resistance surveillance data and the knowledge of the risk factors associated with MDR UTIs in guiding empiric therapy choices.

The initiation of appropriate antimicrobial agents empirically is of utmost importance. However, in order to optimize empiric therapy, local patterns of antimicrobial resistance should be known. Antibiotic resistance rates and patterns differ by patient population type and change over time.^36^ The IDSA 2010 guidelines recommend a 10% to 20% resistance threshold for the treatment of community-acquired UTIs using a particular antibiotic; above this threshold, the agent should not be used for empiric therapy.^6^

In our setting, when compared with other oral agents used for UTIs, isolates were more frequently susceptible to fosfomycin. Fosfomycin susceptibility rates for Enterobacteriaceae were the highest, followed by ciprofloxacin, nitrofurantoin and cefuroxime. Our data confirmed decreased ciprofloxacin susceptibility amongst the Enterobacteriaceae tested. Although fluoroquinolones are highly efficacious in a 3-day regimen, there has been a dramatic increase in resistance to these agents in gram-negative uropathogens in the past decade. A multicentre study conducted between 2007 and 2011 detected a significant decrease in ciprofloxacin susceptibility amongst *E. coli* causing UTIs in both the public and the private sector. Ciprofloxacin susceptibility in the public sector declined by 5% and by 7% in the private sector.^28^ Ongoing indiscriminate and widespread use of ciprofloxacin to treat different infections is expected to cause a further decline in the susceptibility of pathogenic microorganisms to ciprofloxacin. This is particularly concerning, as both the South African Antibiotic Stewardship Programme Adult Antibiotic Guidelines and the Standard Treatment Guidelines and Essential Medicines List for South Africa (SA) recommend ciprofloxacin for management of cystitis and pyelonephritis in patients that are not severely ill and are able to tolerate oral medication.^37,38^ Empirical treatment with ciprofloxacin would be inadequate in almost 30% of patients with UTI at our centre. Therefore, the use of ciprofloxacin at our centre should be reserved for the treatment of UTIs caused by bacteria with known susceptibility to this drug.

Nitrofurantoin susceptibility (70.1%) was lower compared to findings from previous studies in our region, where the reported susceptibility was above 90%.^4,20^ Nitrofurantoin has a reasonable efficacy and a low predisposition to cause collateral damage.^4,28^ Nitrofurantoin is an alternative antibiotic for the treatment of UTIs caused by MDRE, but it is contraindicated in advanced pregnancy and in patients with significant renal dysfunction.^39^

Even though the first-generation cephalosporins reach high concentrations in the urine, they have high resistance rates when compared with other agents.^40^ A study by Chen et al. found a significant decline over a 10-year period for the sensitivity of *E. coli* to first-generation cephalosporins. The sensitivity rate was 72% in 2003 and 28% in 2012.^41^ In 2011, CLSI modified cefazolin breakpoints and this likely contributed to the higher observed resistance rates in this current study. Therefore, this antibiotic cannot be used for empiric UTIs treatment in our institution. The susceptibility of cefuroxime in our study was lower (61.9%) compared to that found by Lewis.^4^ Previous widespread use of cefuroxime for other indications may have contributed to the declined susceptibility.

Although trimethoprim/sulfamethoxazole is still widely used and recommended for acute cystitis therapy, the limitation is that local resistance rates of uropathogens should not exceed 20%, or the infecting strain should be confirmed to be susceptible.^6^ In the present study, the rate of trimethoprim/sulfamethoxazole susceptibility (61.6%) was the lowest amongst the oral antimicrobials tested. A study conducted in South Africa’s Gauteng province similarly found trimethoprim/sulfamethoxazole to be the least efficacious antimicrobial agent against uropathogens causing community-acquired UTIs.^4^ Trimethoprim/sulfamethoxazole has been used as a prophylactic agent against opportunistic infections in people with Acquired Immunodeficiency Syndrome (AIDS) in SA. This may be contributing to the particularly high rates of resistance. Trimethoprim/sulfamethoxazole must be reserved for the treatment of uncomplicated community acquired UTIs where susceptibility of the organism has been confirmed.

Fosfomycin has also been used off-label with success for complicated UTIs excluding pyelonephritis, perinephric abscess or bacteraemic UTIs.^42^ The doses that have been used for complicated UTIs and MDRE vary in literature, for example, 3 g every 48–72 h for 6–21 days has been used. These multi-dose off-label regimens require further microbiological and clinical validation.^43^ Dose adjustment is not required in susceptible groups, such as pregnant women, patients of advanced age, and those with hepatic or renal failure. Therefore, it is a safe drug for patients with hepatic or renal dysfunction or for those receiving concomitant nephrotoxic drugs.^5,44^ Fosfomycin may be used in children; however, the availability of pharmacokinetic data for neonates is very scanty. The dose that has been used for children less than 5 years is 1 g–2 g, but this dose strength is currently not available in SA.^38^

Gardiner et al.^45^ found that the concentration attained by oral fosfomycin in the prostate is sufficient to prevent prostatic infection in patients undergoing transrectal ultrasound-guided biopsy. Fosfomycin may also be considered as an alternative treatment for prostatic infections caused by MDRE.^45^ The usual dosage regimen is 3 g of oral fosfomycin 3 h before and 24 h after traumatic interventions and surgical procedures.^46^ Studies that compared the efficacy of ciprofloxacin versus fosfomycin when used for prophylaxis of urological procedures demonstrated that fosfomycin was associated with lower rates of subsequent UTIs.^47,48^

Research suggests that surgical antibiotic prophylaxis is beneficial for preventing UTIs post-urologic surgeries and procedures.^46^ The selection of the antibiotic used for surgical prophylaxis must also be based on the local epidemiology of antibiotic resistance.^46^ This study did not assess the role of fosfomycin in surgical prophylaxis for urological surgeries and procedures. However, based on the susceptibility of the microorganisms causing UTIs as shown in our research, fosfomycin could be used for surgical prophylaxis in our setting. It is vital to perform local studies to determine the role of fosfomycin above current antibiotic regimens used for surgical prophylaxis.

## Study limitations

This study has several limitations. It was a retrospective single-centre study; therefore, the findings may not be generalised to other institutions with different susceptibility patterns and patient populations. A larger dataset, which includes data from other public and private hospitals, would offer further insight. As CMJAH is a referral hospital, antibiotic resistance rates may be higher than those found in local hospitals. Clinical and Laboratory Standards Institute breakpoints for *E. coli* and *E. faecalis* were extrapolated for the other Enterobacteriaceae and gram-positive organisms, respectively. Confirmatory tests for AmpC-β-lactamase and ESBL production were not performed. Molecular confirmation of carbapenemases production was not performed. Colistin broth microdilution was not performed in this study; therefore, we were not able to comment on the rate of colistin resistance. The small sample size of gram-positives may limit the ability to make reliable conclusions about the activity of fosfomycin against these uropathogens in our setting. This needs to be addressed by performing a larger evaluation. This study was not designed to differentiate between community-acquired and nosocomial UTIs. Lastly, the study focused only on the in vitro susceptibility rates of uropathogens, and was not designed to assess clinical and microbiological outcomes.

## Conclusion

This study is important in that it represents the first local comprehensive evaluation of fosfomycin against gram-negative and gram-positive uropathogens. Fosfomycin has been shown to have good activity against both susceptible and MDR uropathogens. These findings together with its favourable side-effect profile, position it as a potential alternative oral agent for empiric and definitive treatment of UTIs for hospitalised and ambulatory patients at our centre.
